# Beyond pharmacology: The mediating role of expectations in the experience of side effects in anxious patients treated with secukinumab

**DOI:** 10.1016/j.bbih.2026.101376

**Published:** 2026-10-05

**Authors:** Senta Mühlhaus, Frederik Krefting, Daniela Bese, Manfred Schedlowski, Wiebke Sondermann, Stefanie Hölsken

**Affiliations:** a Department of Dermatology, Venereology and Allergology, University Hospital Essen, University of Duisburg-Essen, Essen, Germany; b Institute of Medical Psychology and Behavioral Immunobiology, University Hospital Essen, University of Duisburg-Essen, Essen, Germany; c Department of Clinical Neuroscience, Osher Center for Integrative Medicine, Karolinska Institutet, Stockholm, 171 77, Sweden; d Institute for Medical Education, University Hospital Essen, University of Duisburg-Essen, Essen, Germany; e Department of Dermatology, University Hospital Erlangen, Friedrich-Alexander University Erlangen-Nürnberg, Erlangen, Germany; f Deutsches Zentrum Immuntherapie (DZI), University Hospital Erlangen, Erlangen, Germany

**Keywords:** Psoriasis, Secukinumab, Nocebo effect, Trait anxiety, Treatment expectations

## Abstract

**Introduction:**

Biologics such as the interleukin-17A inhibitor secukinumab have significantly advanced the treatment of psoriasis. However, when discussing treatments, the role of expectations in the perception of side effects is not yet entirely clear. It is known, however, that negative expectations can cause nocebo effects. We investigated whether side-effect expectations mediate the relationship between trait anxiety and the reporting of side effects during secukinumab therapy.

**Methods:**

This post-hoc analysis used data from a randomized controlled trial in adults with moderate-to-severe plaque psoriasis. At the start of the study, trait anxiety and expectations regarding side effects were assessed; self-reported side effects were recorded at weeks 4 and 16. The side-effect outcomes were square-root-transformed prior to analysis to address distributional skew. All mediation steps were estimated using linear regression.

**Results:**

Data from N = 104 participants were analyzed. Higher trait anxiety was associated with more self-reported side effects at week 4, but not at week 16. The association at week 4 was mediated by expectations regarding side effects assessed at baseline.

**Conclusion:**

The results indicate a nocebo effect in more anxious patients in the first weeks of treatment, with anxiety linked to negative treatment expectations which in turn were associated with the experience of side effects. Over time, the level of experienced side effects decreased significantly, potentially due to positive learning experiences occurring over the course of the treatment. Our findings emphasize the significance of targeted patient-centred communication to optimize treatment adherence.

## Highlights

• Mediation analysis reveals negative expectations as the mechanism linking trait anxiety to side effect perception.
• Trait anxiety drives early nocebo responses in biological therapy.
• The anxiety-side effect association diminishes over the course of treatment.
• Positive treatment experience may counteract nocebo effects.

## Graphical abstract

Negative treatment expectations, rather than anxiety itself, were found to correlate with the experience of side effects in psoriasis patients receiving biologic treatment with secukinumab. These findings highlight nocebo mechanisms in systemic therapy and stress the importance of targeted doctor–patient communication to reduce harm while enhancing adherence and safety. Image created in BioRender. Mühlhaus, S. (2026) https://BioRender.com/jeb7329.

**Figure:**
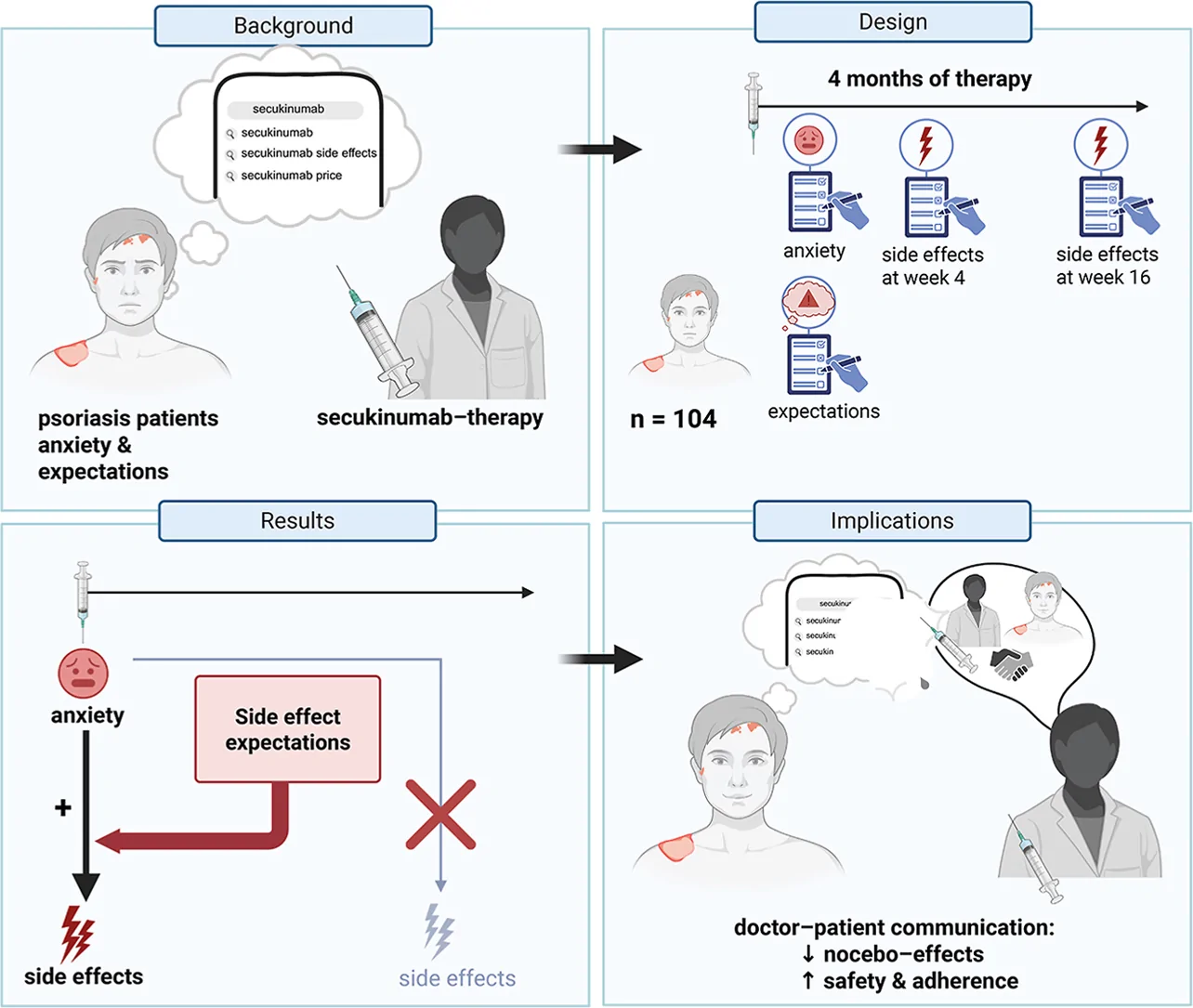

## 1 Introduction

### 1.1 Background

Psoriasis is currently understood as a chronic, systemic, immune-mediated inflammatory disease affecting three main domains - the skin, the joints and bones, and the blood vessels, all characterized by a similar pattern of inflammation (Mrowietz et al., 2024). The global prevalence is around 2 %, with no gender predominance (Christophers, 2001). The most common type, plaque psoriasis, typically presents with well-demarcated, erythematous plaques covered with silvery scales (Boehncke and Schön, 2015). These skin manifestations, together with the associated itching, cause a great burden for those affected as reflected in a significant reduction in quality of life (QoL) (Finlay and Coles, 1995).

Therapeutic strategies for psoriasis have evolved significantly over the past decades (Reid and Griffiths, 2020). Modern systemic treatments have been shown to substantially reduce symptom burden and improve QoL, while mostly being well tolerated (Sondermann and Körber, 2018). For instance, clinical trials of secukinumab, a monoclonal antibody targeting interleukin (IL) 17A, could show 90 % improvement in the Psoriasis Area and Severity Index (PASI) after 12 weeks in 54 % of the patients (Thaçi et al., 2015).

While treatment can nowadays successfully reduce patients’ burden regarding skin symptoms, psoriasis is associated with several comorbidities, which also need to be managed to ensure appropriate care. These comorbidities include an increased risk for metabolic syndrome, cardiovascular disease, depression and suicidal ideation (Armstrong et al., 2025; Hölsken et al., 2021; Masson et al., 2020). Furthermore, patients with psoriasis exhibit elevated rates of anxiety disorders relative to healthy controls, with severe psoriasis increasing the incidence by 43 % (Kurd et al., 2010). This elevation was found to concern both state and trait anxiety (Knowles and Olatunji, 2020). State anxiety can be defined as a transient emotional state that is elicited by specific (subjectively) threatening situations. Trait anxiety, in contrast, represents a stable tendency to react anxiously to various stressors. This tendency is shaped by factors such as gender, culture and age (Reiss, 1997).

Besides being a burden itself, anxiety may modulate the outcomes of anti-psoriatic treatments by shaping patients’ treatment expectations and thus placebo and nocebo effects. The term placebo or nocebo effect denotes an improvement or worsening of symptoms that cannot be pharmacologically explained but rather be attributed to factors inherent to the patient or clinical context (Wager and Atlas, 2015). Anxiety has been identified as a driver of side-effect expectations (Basedow et al., 2023) and as a significant amplifier of nocebo responses, e.g. in patients suffering from pain (Corsi and Colloca, 2017). So far, however, this connection has received only little recognition in dermatological research, which may contribute to an underestimation of these mechanisms in the field (Sonthalia et al., 2015).

In pivotal studies for anti-psoriatic medication, adverse events were found to be reported in around 50 % of patients randomized to the placebo groups despite not receiving active medication, pointing to substantial nocebo effects (Ma et al., 2024). Negative expectations may thereby result in reduced adherence and discontinuation of treatment. Therefore, identifying patients with an increased risk to experience nocebo effects is of high clinical importance (Frisaldi et al., 2024).

This association may be of particular significance in the context of biologic therapy for psoriasis. Patients eligible for such treatment are typically those with a high disease severity and significantly impaired quality of life. They therefore represent a subgroup with particularly elevated rates of anxiety (Fleming et al., 2017), suggesting that the study of this population involves an implicit pre-selection of anxiety-prone individuals. Furthermore, biologics are administered via subcutaneous injection, and it has been shown that invasive treatment methods elicit stronger treatment expectations and more pronounced placebo effects compared to non-invasive alternatives (Rief and Wilhelm, 2024). Whether invasive administration similarly amplifies negative expectations and the perception of side effects has received significantly less attention to date. The context of biologic therapy therefore offers a particularly insightful framework for investigating anxiety-related nocebo effects.

If trait anxiety fosters negative expectations regarding treatment, and these expectations in turn amplify the perception of side effects, then expectations could act as a mediating mechanism through which anxiety influences nocebo reactions. Recent meta-analytic evidence has shown that situationally induced expectations and state anxiety reliably predict nocebo effects, whereas trait anxiety shows no direct association (Rooney et al., 2023). This raises the question of whether trait anxiety may exert its influence indirectly via negative treatment expectations.

Examining this mediation pathway would not only clarify the psychological mechanisms underlying nocebo effects in clinical settings, but also identify expectations as a potentially modifiable variable for clinical interventions.

### 1.2 Objectives

In this study, we aimed to assess the potential association between trait anxiety and negative treatment expectations in patients initiating biologic treatment with the IL-17A blocker secukinumab. We hypothesized that patients with heightened levels of anxiety would be more likely to expect side effects and, in turn, be more likely to actually experience side effects.

## 2 Methods

### 2.1 Study design

This study was a post-hoc analysis of data from a randomized, controlled proof-of-concept study, investigating expectation modulation in patients with psoriasis undergoing therapy with the IL-17A inhibitor secukinumab at Essen University Hospital from October 2020 until November 2023 (Hölsken et al., 2025). The present analyses were not pre-registered and should be considered exploratory. The parent trial was prospectively registered in the German Clinical Trials Register (DRKS00022104). The parent trial comprised three groups: a treatment as usual group (300 mg secukinumab), a dose control group (75 % dose reduction) and an experimental group (75 % dose reduction and expectation modulation via positive cover story).

The present analysis used longitudinal questionnaire data, specifically those assessing trait anxiety and side effects. Clinical outcomes of the parent trial and the effectiveness of the expectation manipulation not the focus of this analysis. Data from all three groups were pooled, as the parent trial did not demonstrate significant expectation modulation across groups (Hölsken et al., 2025).

The dose group (300 mg vs. reduced dose) was included a priori as a covariate in all mediation analyses, as it represents a previously defined randomization factor in the parent randomized trial and may independently affect pharmacological side-effect burden (Raab et al., 2000).

For other potential confounders (age, sex, body mass index in kg/m^2^ (BMI), PASI, DLQI), Spearman correlations with the key study variables were computed to evaluate classical confounding criteria (see Supplementary Table S2). No variable met criteria for confounding. Age showed a significant correlation with experienced side effects at week 4 (r = −0.32, p = .001) but not with trait anxiety (r = −0.10, p = .292), qualifying it as a precision covariate (VanderWeele, 2019). To assess the robustness of the primary findings, it was therefore included as a covariate in a sensitivity analysis (Thabane et al., 2013) (see Supplementary Table S3). Patients or members of the public were not involved in the design, conduct, reporting, or dissemination of this study. The study was approved by the Ethics Committee of the Medical Faculty at the University of Duisburg-Essen (IRB protocol number 19-8636-BO). All participants provided written informed consent for study participation and data processing prior to study participation.

### 2.2 Participants and eligibility criteria

N = 120 patients with moderate-to-severe plaque psoriasis were recruited via the dermatology outpatient clinic at Essen University Hospital, advertisements at private practices, and social media. Patients were eligible for participation if they had a PASI of at least 12 and a Body Surface Area (BSA) affected by psoriasis of at least 10 %. Additional inclusion and exclusion criteria were derived from the summary of product characteristics for secukinumab, including standard contraindications to biologic therapy.

### 2.3 Data collection

Data included in this study was assessed at treatment onset as well as after one and four months of secukinumab treatment when patients had received five or nine injections respectively. Patient reported data were collected using standardized questionnaires, which were completed by the patients before receiving their secukinumab injection. Psoriasis severity was assessed by trained dermatologists who were blinded regarding the study procedures. Data was pseudonymized using the software ALIIAS (Englert et al., 2023).

### 2.4 Variables

The State-Trait Anxiety and Depression Inventory (STADI) was employed to measure trait anxiety at treatment onset (Laux et al., 2013). The trait anxiety score is calculated as the sum of two subscales: emotionality and worry. Expectations regarding the treatment were measured with the Treatment Expectation Questionnaire (TEX-Q) at two timepoints: at study enrolment and one week after the first injection of secukinumab. The questionnaire comprises six subscales assessing different aspects of expectations with a total of 15 items (Shedden-Mora et al., 2023). For the purpose of this study, only the side effects subscale was used as prior research proposes a specific influence of this construct in the studied context.

Perceived side effects were assessed through patient self-report on an 11-point scale using a subscale of the Generic Rating Scale for Treatment Experiences, Expectations, and Effects (GEEE) (Rief et al., 2021) both after 4 and 16 weeks of treatment. It should be noted that adverse events were included as patient self-report only. Numbers of adverse events that were clinically suspected to be treatment-related were low (see (Hölsken et al., 2025). The employed approach thus captures the subjective perception of side effects but does not reflect clinically verified adverse events.

For the purpose of sample characterization, psoriasis severity at treatment onset was assessed using the Dermatology Life Quality Index (DLQI) and PASI. The DLQI assesses disease-related QoL over the past seven days using ten questions covering symptom impact on daily activities, work, relationships, and treatment side effects (Finlay and Khan, 1994). Next to these patient-reported variables, the PASI was assessed as an objective marker of the disease (Nast et al., 2021).

### 2.5 Statistical analysis

Of the 120 enrolled patients, 15 were lost to follow-up prior to data collection (missed appointments: n = 9; COVID-19 quarantine: n = 2; discontinued intervention: n = 2; suspected cancer: n = 1; deceased: n = 1), as documented in the study flowchart (Fig. 1).

**Fig. 1:**
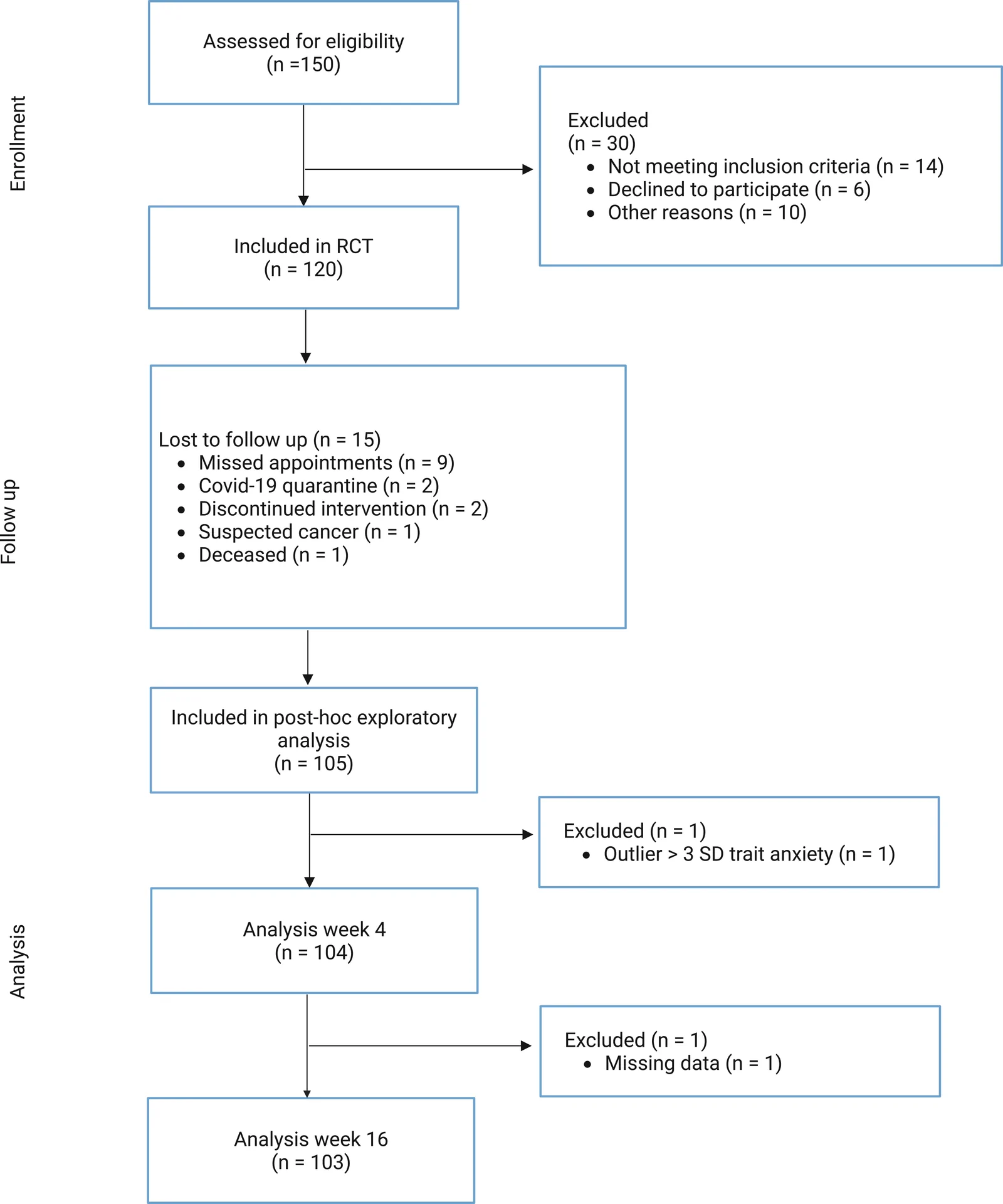
Study flowchart describing the number of patients included in analyses.

One additional participant was excluded due to trait anxiety scores exceeding three standard deviations above the sample mean, yielding a final analytic sample of N = 104. A sensitivity analysis retaining this participant is reported in Supplementary Table S1. The additional analysis shows no relevant impact of this individual participant on the reported results. Missing data were handled by pairwise deletion. The analytic sample therefore varies slightly across analyses (N = 102 - 104).

Participants with missing values on the key variables (trait anxiety, expectations, experienced side effects) were excluded from the respective analyses. No imputation procedures were applied.

All analyses were performed using R Statistical Software (version 4.2.2 (Team, 2022)).

The GEEE side-effect score showed pronounced positive skew with substantial zero-inflation (64.4 % of participants scoring zero at week 4). To address this distributional property while retaining the full range of the continuous scale, experienced side effects were square-root-transformed prior to analysis. Square root transformation is an established approach for right-skewed, zero-inflated data that reduces the influence of extreme values without the information loss associated with dichotomization. All mediation models were therefore estimated using linear regression on the square-root-transformed GEEE scores.

R^2^ was reported per mediation step as a measure of model fit, and Cohen's f^2^ was calculated for the full model (Step 4) at both timepoints as an overall effect size measure.

The mediation analysis included four stages to examine the relationship between trait anxiety (STADI), expectations of side effects (TEX-Q) and perception of side effects (GEEE) (Baron and Kenny, 1986).

First, a linear regression was run to assess the total effect of trait anxiety on square-root-transformed experienced side effects (path c), separately for week 4 and week 16.

Second, a linear regression was calculated to analyze the effect of trait anxiety on side-effect expectations (path a).

Third, a further linear regression was performed to assess the effect of expectations on experienced side effects (path b).

Finally, a multiple linear regression was conducted to assess the direct effect of trait anxiety on side-effect experience after controlling for expectations (path c').

Complete mediation was inferred when the total effect (path c) was statistically significant and the direct effect (path c') was rendered non-significant after inclusion of the mediator. Dose group (300 mg vs. reduced dose) was included as a covariate in all four steps.

Model assumptions were checked for all regression models using the Shapiro-Wilk test for normality of residuals, the Breusch-Pagan test for homoscedasticity, and variance inflation factors (VIF) for multicollinearity. Detailed diagnostic results are reported in Supplementary Table S5.

To examine temporal changes in expectations and side effects over the study period, two paired t-tests were conducted: one comparing side-effect expectations at study enrolment and one week after the first injection of secukinumab, and one comparing experienced side effects at week 4 and week 16. Effect sizes were calculated as Cohen's d.

Supplementary mediation analyses using state anxiety prior to treatment as predictors are reported in Supplementary Table S4.

The de-identified dataset supporting these findings is openly available in the Open Science Framework (OSF) at https://osf.io/acufz/files/osfstorage/68de7ffbed4191d3b2fd0493. The R code used for the analyses is available from the corresponding author upon reasonable request.

## 3 Results

### 3.1 Sample characteristics

A total of N = 104 patients were included in the analysis, of whom 36 were female (35 %) and 68 male (65 %). The age range was 20 to 74 years, with a mean age of 46 years. Sample characteristics are shown in Table 1.

**Table 1:** Descriptive statistics.

	N	Mean	SD	Min	Max
Baseline DLQI	104	12.65	6.90	0	27
Baseline PASI	104	17.75	5.36	11.2	34
Trait anxiety	104	19.52	4.98	11	31
Age (years)	104	46.38	14.82	20.66	74.36
BMI (kg/m^2^)	103	29.36	7.63	18.21	51.32
Baseline side-effect expectations	104	3.07	1.94	0	8.33
Side-effect expectations (week 1)	102	2.53	1.72	0	8
Side-effect experience at week 4	104	1.14	1.71	0	8
Side-effect experience at week 16	103	0.78	1.40	0	7

Spearman correlation analyses (Supplementary Table S2) confirmed that no demographic or clinical variable (age, sex, BMI, PASI, DLQI) correlated with both trait anxiety and experienced side effects. Age showed a significant correlation with experienced side effects in week 4 (r = −0.32, p = .001) and was included in a sensitivity analysis, also reported in Supplementary Table S3. The inclusion of age as a covariate did not change the mediation pattern.

### 3.2 Side effects after 4 weeks

A mediation analysis was performed to assess the role of side-effect expectations in the association between trait anxiety and experienced side effects at week 4 (see Table 2). There was a statistically significant total effect (path c), with a one-unit increase in trait anxiety associated with a 0.04-unit increase in the square-root-transformed side-effect score (t = 2.74, p = .007, β = 0.04, 95 % CI [0.01, 0.08]). Dose group emerged as a significant covariate in step 1 at week 4 (β = 0.34, p = .046), indicating that the reduced-dose group reported higher side-effect scores at week 4.

**Table 2:** Results of Mediation Analysis for week 4 and week 16.

Timepoint	Step	Predictor	β	SE	t	p	R^2^
Week 4	Step 1 (c)	Trait Anxiety	0.04	0.02	2.74	.007	.092
	Step 2 (a)	Trait Anxiety	0.13	0.04	3.60	<.001	.117
	Step 3 (b)	Expectations baseline	0.15	0.04	3.74	<.001	.144
	Step 4 (c')	Trait Anxiety	0.03	0.02	1.67	.098	.167
	Step 4 (b')	Expectations baseline	0.12	0.04	2.99	.003	.167
Week 16	Step 1 (c)	Trait Anxiety	0.02	0.02	1.59	.116	.025
	Step 2 (a)	Trait Anxiety	0.13	0.04	3.60	<.001	.117
	Step 3 (b)	Expectations baseline	0.13	0.04	3.75	<.001	.124
	Step 4 (c')	Trait Anxiety	0.01	0.02	0.34	.735	.125
	Step 4 (b')	Expectations baseline	0.13	0.04	3.36	.001	.125

β = unstandardized regression coefficient; SE = standard error; R^2^ = coefficient of determination. Expectations = TEX-Q side effect expectations subscale. Dose group was included as a covariate in all models.

The first part of the indirect effect (path a), examining the association between trait anxiety and side-effect expectations, was statistically significant (t = 3.60, p < .001, β = 0.13, 95 % CI [0.06, 0.21]), indicating that a one-unit increase in trait anxiety was associated with a 0.13-point increase in expected side effects.

The second part of the indirect effect (path b) was likewise significant: baseline side-effect expectations significantly predicted experienced side effects at week 4 (t = 3.74, p < .001, β = 0.15, 95 % confidence interval (CI) [0.07, 0.23]). This association is illustrated in Fig. 2.

**Fig. 2:**
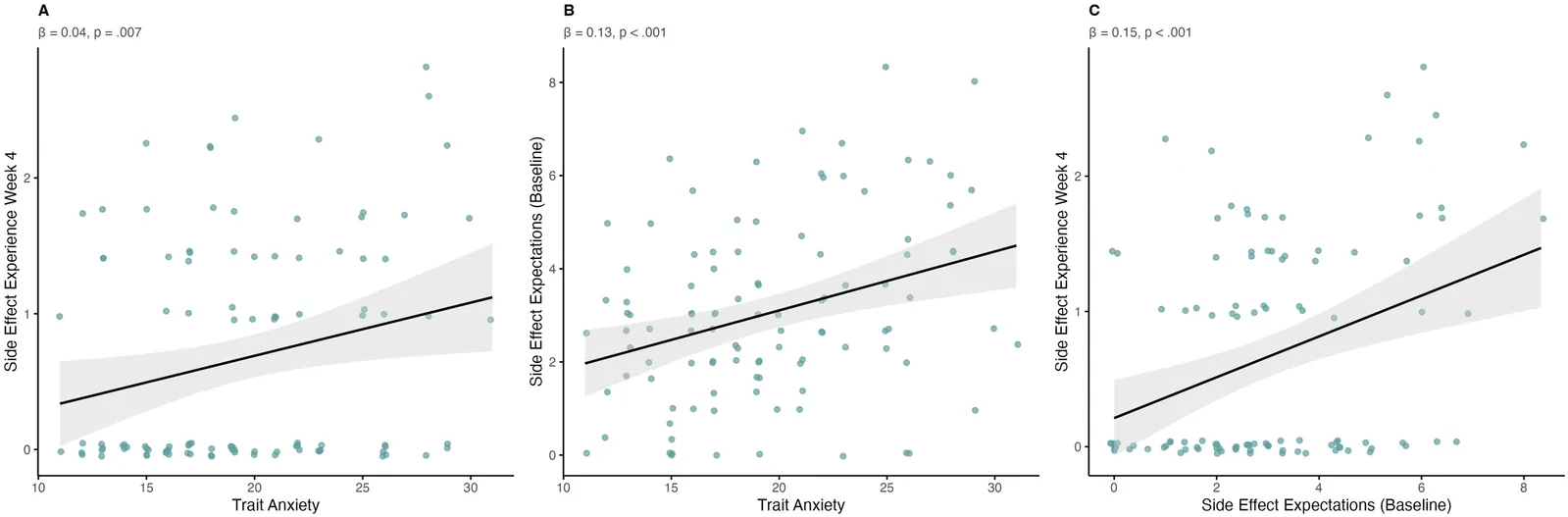
Scatter plots of the data underlying the key mediation paths at week 4. A) Association between trait anxiety and experienced side effects at week 4, with side effect experience given as square-root-transformed scores of the Generic Rating Scale for Treatment Experiences, Expectations, and Effects (GEEE). B) Association between trait anxiety and side effect expectations prior to treatment initiation. C) Association between side effect expectations and experienced side effects at week 4 (again, GEEE scores underwent square-root-transformation). Each panel includes a linear regression line with 95 % confidence band.

When both trait anxiety and expectations were included in the model, expectations remained a significant predictor of experienced side effects (path b': t = 2.99, p = .003, β = 0.12, 95 % CI [0.04, 0.21]), while the direct effect of trait anxiety became non-significant (path c': t = 1.67, p = .098, β = 0.03, 95 % CI [−0.01, 0.06]), indicating full mediation. The full model explained 16.7 % of the variance in experienced side effects at week 4 (R^2^ = .167, Cohen's f^2^ = 0.20), corresponding to a medium effect size. Fig. 3 summarizes the mediation model.

**Fig. 3:**
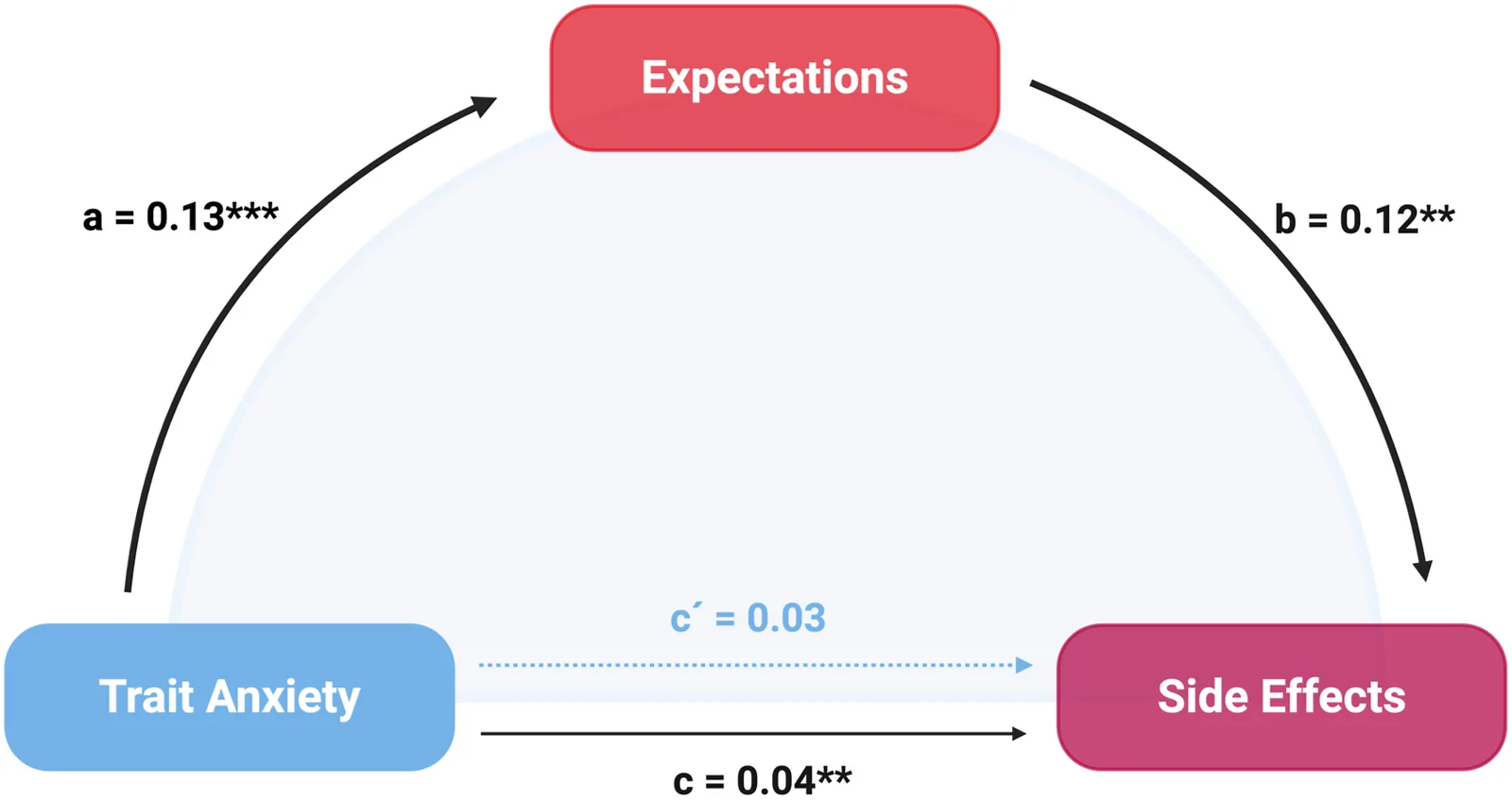
Mediation model of the association between trait anxiety and side effect experience after four weeks of treatment, with side effect expectations as a mediator. All paths estimated using linear regression with square-root-transformed GEEE side effect experience as outcome. c = total effect; c' = direct effect controlling for expectations; a = effect of trait anxiety on expectations; b = effect of expectations on side effects. Full statistics including p-values are reported in Table 2. *p < .05, **p < .01, ***p < .001.

### 3.3 Side effects after 16 weeks

In contrast to week 4, there was no significant total effect (path c) of trait anxiety on experienced side effects at week 16 (t = 1.59, p = .116, β = 0.02, 95 % CI [−0.01, 0.05]). As the first criterion for formal mediation was not met, complete mediation could not be established. Nevertheless, all 4 steps of the mediation model were estimated for completeness (see Table 2). Path a, i.e., the association between trait anxiety and side-effect expectations, was identical to the mediation model for week 4. Baseline side-effect expectations significantly predicted experienced side effects at week 16 (path b: t = 3.75, p < .001, β = 0.13, 95 % CI [0.06, 0.20]), and remained significant after controlling for trait anxiety (path b': t = 3.36, p = .001, β = 0.13, 95 % CI [0.05, 0.20]). The direct effect of trait anxiety was likewise non-significant after controlling for expectations (path c': t = 0.34, p = .735, β = 0.01, 95 % CI [−0.03, 0.04]). The full model (Step 4) explained 12.5 % of the variance in experienced side effects at week 16 (R^2^ = .125, Cohen's f^2^ = 0.14), corresponding to a small to medium effect size.

Model diagnostics for all mediation models are reported in Supplementary Table S5. Multicollinearity was not a concern in any of the analyses (all VIFs < 1.17). At week 4, assumptions of homoscedasticity were met. Following the Shapiro-Wilk test, assumptions regarding normality of residuals were not met for square-root-transformed GEEE scores. However, given the sample size of N = 104, coefficient estimates remain unbiased under the central limit theorem. At week 16, heteroscedasticity was detected in the models involving the GEEE outcome (Breusch-Pagan: p = .003).

### 3.4 Change of expectation of side effects and experienced side effects

To examine whether side-effect expectations and experienced side effects changed over the course of the study, two paired t-tests were conducted. Side-effect expectations decreased significantly from baseline (M = 3.07, SD = 1.94) to after the first injection of secukinumab (week 1: M = 2.53, SD = 1.72), t (101) = 3.10, p = .003, d = 0.31. Similarly, experienced side effects decreased significantly from week 4 (M = 1.14, SD = 1.71) to week 16 (M = 0.78, SD = 1.40), t (102) = 2.43, p = .017, d = 0.24.

## 4 Discussion

### 4.1 Overview

In the present study, patients with psoriasis were followed over a period of four months of secukinumab therapy. During that time, their anxiety, side-effect expectations and actual experience of side effects were assessed to explore potential links. Specific studies on expectations and side effects of biologic therapies in psoriasis are rare. A first qualitative study (Hölsken et al., 2024) examined patients’ perception of secukinumab treatment. The interviewed patients had high expectations regarding efficacy, but a significant portion also reported starting treatment with the expectation to experience side effects. The aim of our study was to elaborate upon the underlying factors of these side-effect expectations, as well as their potential effects.

### 4.2 Principal findings

We found that psoriasis patients with higher trait anxiety at treatment onset were more likely to report side effects after four weeks of treatment. This association between trait anxiety and self-reported side effects was fully mediated by side-effect expectations. This suggests that negative expectations are associated with the increased perception of side effects, complementing findings from earlier studies (Krefting et al., 2022). For example, a recent study by Frisaldi and colleagues demonstrated state anxiety and negative expectations to be associated with the occurrence of expected side effects in patients with myasthenia gravis (Frisaldi et al., 2024). Using a mediation model, our study elaborates upon the suggested underlying pathways and extends prior findings to a larger cohort of patients suffering from psoriasis.

### 4.3 Interpretation

Interestingly, we only saw an association of trait anxiety and side-effect experience after four weeks of treatment and not after 16. From a statistical point of view, this change in association is consistent with a reduction in perceived side effects over the course of treatment. Similar reductions in side-effect perception were also seen in a range of earlier studies, for example in samples of breast cancer patients (Van Esch et al., 2011) or patients suffering from rheumatoid arthritis (Nestoriuc et al., 2010). A meta-analysis investigating adverse reactions to the COVID-19 vaccine found a significant decrease in side effects from the first to the second injection in placebo recipients, while this decrease was not present in verum group participants (Haas et al., 2022). To explain this reduction in side effects Haas et al. claimed that high anticipatory anxiety fostered early side effects and this anxiety habituated over time. However, in our study, we used trait anxiety as a measure - a dispositional and thus more stable one (Spielberger et al., 1983). Our results therefore suggest a shift in expectations rather than one in trait anxiety to be associated with the observed reduction.

In line with this interpretation, side-effect expectations decreased significantly, providing preliminary evidence for a change in expectations early in the treatment course. While our data do not allow for a direct test of a causal influence on the attenuation of the trait anxiety-side effect association and should therefore be interpreted cautiously, they point to a potentially promising target for further investigation.

While the employed mediation model implies a sequential relationship of anxiety and expectations, other models assume a more parallel processing. In their meta-analysis, Rooney et al. (2023) suggest expectations and anxiety to represent distinct pathways through which treatment information shapes nocebo responses, with expectations reflecting a cognitive appraisal and anxiety an affective response. Future experimental studies should aim to disentangle these pathways to better understand the underlying mechanisms.

### 4.4 Possible mechanisms and implications

But why would this shift occur? Consistent with the outcomes reported in the parent trial, patients experienced significant improvements across all assessed disease severity measures over the course of treatment, as reported in the clinical outcomes of the parent trial (Hölsken et al., 2025). At the same time, overall numbers of reported side effects were low. This combination of symptom improvement alongside the absence of serious side effects may have provided the corrective information necessary for patients to update their initial negative expectations.

To understand why such a corrective experience might be particularly relevant in this context, it is important to consider how anxiety and negative expectations interact.

Anxiety can be both cause and consequence of negative expectations (Grupe and Nitschke, 2013). High anxiety scores of patients prior to therapy could have led to hypervigilance and somatization, with patients misinterpreting normal bodily sensations as serious side effects or converting psychological distress into physical symptoms. This in turn prevents corrective experiences and contributes to the person concerned remaining in a stressful state of constant worry. However, as the data from our study suggest, positive learning experiences like receiving effective and safe medication may work as an ‘expectation update’.

### 4.5 Strengths and limitations

There are only a few studies directly investigating the mediation pathway: although it has been theorized that expectation is the missing link between anxiety and symptom report, (Bingel, 2020; Price et al., 1999)^,^ empirical evidence for this mediation is rare and mainly investigated in painful conditions rather than chronic inflammatory diseases. (Rubanets et al., 2024; Whitford and Olver, 2012)^,^ Our study possesses several methodological and conceptual strengths. Firstly, we were able to investigate the mediating influence of expectations on side effects in a large clinical cohort. Secondly, the study used a relatively long follow-up period, allowing us to examine whether the observed associations between trait anxiety, expectations, and side effects remained stable or differed across two treatment timepoints. With this, our study is the first to show that this association can also be observed in the field of dermatology - specifically in a chronic inflammatory skin disease.

Regarding the different facets of treatment expectations, we decided to solely focus on side-effect expectations, as they present the most straightforward potential link between anxiety and side-effect experience. Using additional subscales would have introduced conceptually distinct constructs and inflated the number of comparisons. Nevertheless, it might be worthwhile in future studies to include other subscales, for example to assess a potential beneficial effect of positive treatment expectations in this framework.

However, certain limitations to the design also need to be considered. Most importantly, this study was a post-hoc exploration of data from a larger randomized controlled trial (RCT). Therefore, its results should be carefully confirmed in a pre-registered design. Since we did not continuously measure expectations over the course of the study, we cannot analyze if changed expectations explain the lack of association between trait anxiety and side effects after 16 weeks. Further studies therefore should consider a repeated assessment to test this hypothesis. Given the observational design of our study, our mediation model represents theoretical assumptions about possible dependency structures, rather than proven relationships. Nevertheless, the assessment of the predictor variables before the start of treatment and the side effects during treatment allows a plausible temporal modeling of the relationships.

Due to this study being part of a larger RCT, two-thirds of assessed patients received a reduced dosage of secukinumab, potentially leading to lower side effect burden.

While our study showed that side-effect expectations and experiences are an important topic even in treatments with a good safety profile, statistical analyses were clearly limited by floor effects in the form of low side-effect expectations and low numbers of experienced side effects.

The low mean side effect ratings observed in our sample are consistent with the well-established safety profile of secukinumab (Gottlieb et al., 2022).

Nevertheless, we cannot rule out a drop-out bias. Of the 15 participants lost to follow-up, ten had no data on any study variable and were therefore excluded entirely. The remaining five had baseline data only. As no follow-up outcome data were available for these individuals, a formal test of drop-out bias was not feasible, and the possibility that more anxious participants were disproportionately likely to withdraw cannot be set aside.

Additionally, while trait anxiety was chosen as the primary predictor due to its stable dispositional nature, allowing for more generalizable conclusions about vulnerability to nocebo effects, supplementary analyses revealed that state anxiety showed a comparable mediation pattern. The relative contributions of trait and state anxiety therefore remain unclear and warrant systematic investigation in future studies.

## 5 Conclusion

Our study adds important knowledge for the fields of placebo and psychodermatology research. We saw further evidence that trait anxiety and negative expectations may play a role in experiencing side effects during modern psoriasis therapy. Moreover, our findings highlight the potential role of trait anxiety in the formation of nocebo effects. The finding that the initial association between trait anxiety and side effects declines over the course of treatment suggests that nocebo effects are dynamic and may be modulated by positive experience.

If confirmed in prospective trials, our findings carry potential implications for clinical practice. Trait anxiety can be assessed with validated instruments at treatment initiation, providing a straightforward screening opportunity to identify patients at elevated risk of nocebo-related side effects. Palese et al. (2019) demonstrated that nurses are aware of contextual factors that can prevent nocebo effects, yet these are inconsistently implemented in routine care. The present findings provide preliminary support for expectation-focused communication as a potentially promising intervention: clinicians and nurses caring for patients with high trait anxiety should proactively address negative expectations, e.g. providing information on the treatment's safety profile.

Future research should focus on how patients with high trait anxiety and a tendency towards nocebo effects can be better treated and cared for. Especially regarding expensive biologic therapies such as secukinumab, it is of crucial importance to minimize nocebo effects in order to avoid treatment discontinuations and increase patients’ quality of life. This could be achieved through targeted patient education and personalized communication with the aim of addressing negative expectations at an early stage.

## CRediT authorship contribution statement

**Senta Mühlhaus:** Writing – review & editing, Supervision, Resources, Funding acquisition, Conceptualization. **Frederik Krefting:** Writing – review & editing, Investigation. **Daniela Bese:** Writing – review & editing, Investigation. **Manfred Schedlowski:** Writing – original draft, Visualization, Software, Methodology, Formal analysis, Data curation. **Wiebke Sondermann:** Writing – review & editing, Supervision, Resources, Project administration, Conceptualization. **Stefanie Hölsken:** Writing – review & editing, Software, Methodology, Formal analysis, Data curation, Conceptualization.

## Ethics declaration

Written informed consent to take part in the study and to publish the article has been obtained from all participants or their legal representatives prior to study inclusion. The privacy rights of participants have been observed.

This study was performed in compliance with relevant laws, regulatory frameworks and guidelines where the research took place. This study was conducted in accordance with the Declaration of Helsinki. This study was approved by the Ethics Committee of the Medical Faculty at the University of Duisburg-Essen (Approval No. 19-8636-BO).

## Funding

Gefördert durch die Deutsche Forschungsgemeinschaft (DFG)—Projektnummer 422744262—TRR 289—Funded by the Deutsche Forschungsgemeinschaft (DFG, German Research Foundation)—Project-ID 422744262—TRR 289.

## Declaration of competing interest

The authors declare the following financial interests/personal relationships which may be considered as potential competing interests: Wiebke Sondermann, Manfred Schedlowski reports financial support was provided by German Research Foundation. Wiebke Sondermann reports a relationship with medi GmbH und Co KG that includes: funding grants. Wiebke Sondermann reports a relationship with AbbVie Deutschland GmbH & Co KG that includes: speaking and lecture fees and travel reimbursement. Manfred Schedlowski, Frederik Krefting reports a relationship with AbbVie Deutschland GmbH & Co KG that includes: speaking and lecture fees. Wiebke Sondermann reports a relationship with Almirall Hermal GmbH that includes: funding grants, speaking and lecture fees, and travel reimbursement. Manfred Schedlowski reports a relationship with Almirall Hermal GmbH that includes: speaking and lecture fees. Frederik Krefting reports a relationship with Almirall Hermal GmbH that includes: consulting or advisory and funding grants. Wiebke Sondermann reports a relationship with Amgen GmbH that includes: speaking and lecture fees. Wiebke Sondermann reports a relationship with Bristol-Myers Squibb GmbH und Co KGaA that includes: speaking and lecture fees and travel reimbursement. Manfred Schedlowski reports a relationship with Bristol-Myers Squibb GmbH und Co KGaA that includes: speaking and lecture fees. Frederik Krefting reports a relationship with Bristol-Myers Squibb GmbH und Co KGaA that includes: travel reimbursement. Wiebke Sondermann reports a relationship with Celgene GmbH that includes: speaking and lecture fees. Wiebke Sondermann reports a relationship with GSK that includes: speaking and lecture fees. Wiebke Sondermann reports a relationship with Janssen-Cilag GmbH that includes: speaking and lecture fees and travel reimbursement. Manfred Schedlowski reports a relationship with Janssen-Cilag GmbH that includes: speaking and lecture fees. Wiebke Sondermann reports a relationship with LEO Pharma that includes: speaking and lecture fees and travel reimbursement. Frederik Krefting reports a relationship with LEO Pharma that includes: consulting or advisory. Wiebke Sondermann reports a relationship with Lilly Deutschland GmbH that includes: speaking and lecture fees and travel reimbursement. Manfred Schedlowski reports a relationship with Lilly Deutschland GmbH that includes: speaking and lecture fees. Frederik Krefting reports a relationship with Lilly Deutschland GmbH that includes: travel reimbursement. Wiebke Sondermann reports a relationship with MSD Sharp & Dohme GmbH that includes: speaking and lecture fees and travel reimbursement. Wiebke Sondermann reports a relationship with Novartis that includes: funding grants, speaking and lecture fees, and travel reimbursement. Frederik Krefting reports a relationship with Novartis that includes: travel reimbursement. Wiebke Sondermann reports a relationship with Pfizer that includes: speaking and lecture fees. Wiebke Sondermann reports a relationship with Roche that includes: speaking and lecture fees. Wiebke Sondermann reports a relationship with Sanofi-Aventis Deutschland GmbH that includes: speaking and lecture fees and travel reimbursement. Wiebke Sondermann reports a relationship with Takeda Pharma Vertrieb GmbH & Co KG that includes: speaking and lecture fees. Wiebke Sondermann reports a relationship with UCB Pharma GmbH that includes: speaking and lecture fees and travel reimbursement. Frederik Krefting reports a relationship with UCB Pharma GmbH that includes: travel reimbursement. Frederik Krefting reports a relationship with Johnson & Johnson that includes: consulting or advisory, speaking and lecture fees, and travel reimbursement. Frederik Krefting reports a relationship with Boehringer Ingelheim Pharma GmbH & Co KG that includes: travel reimbursement. Manfred Schedlowski reports a relationship with Berlin-Chemie AG that includes: speaking and lecture fees. Frederik Krefting reports a relationship with Galderma that includes: consulting or advisory. Manfred Schedlowski reports a relationship with Mylan Germany GmbH that includes: speaking and lecture fees. If there are other authors, they declare that they have no known competing financial interests or personal relationships that could have appeared to influence the work reported in this paper.

## Data Availability

The data that support the findings of this study are openly available in the OSF at https://osf.io/acufz/files/osfstorage/68de7ffbed4191d3b2fd0493.
