# Supplementary material for: Beyond pharmacology: The mediating role of expectations in the experience of side effects in anxious patients treated with secukinumab
Source: Brain Behav Immun Health. 2026 Oct 5;58:101376. doi: 10.1016/j.bbih.2026.101376 (PMC13683029; doi:10.1016/j.bbih.2026.101376)
Supplement: Supplementary file 1 [file mmc1.docx]

Supplementary

**Table S1**

Sensitivity analysis including the participant excluded from the primary analysis due to trait anxiety scores exceeding three standard deviations above the sample mean. Unstandardized regression coefficients (β) and p-values are shown for the four-step Baron and Kenny (1986) mediation model at Week 4 and Week 16, comparing the primary analytic sample (N = 104) with the full sample (N = 105). Dose group (300 mg vs. reduced dose) was included as a covariate in all steps.

| **Timepoint** | **Step** | **Path** | **Main β (N=104)** | **Main p** | **Sensitivity β (N=105)** | **Sensitivity p** |
| --- | --- | --- | --- | --- | --- | --- |
| Week 4 | 1 - Total effect (c) | Trait anxiety → Side effects | 0.04 | .007 | 0.033 | .030 |
| Week 4 | 2 - Path a | Trait anxiety → Expectations | 0.13 | < .001 | 0.095 | .009 |
| Week 4 | 3 - Path b | Expectations → Side effects | 0.30 | .008 | 0.15 | <.001 |
| Week 4 | 4 - Direct effect (c') | Trait anxiety → Side effects | 0.03 | 0.098 | 0.02 | .176 |
| Week 4 | 4 - Path b' | Expectations → Side effects | 0.12 | 0.003 | 0.136 | .001 |
| Week 16 | 1 - Total effect (c) | Trait anxiety → Side effects | 0.02 | 0.116 | 0.017 | .223 |
| Week 16 | 2 - Path a | Trait anxiety → Expectations | 0.13 | <.001 | 0.095 | .009 |
| Week 16 | 3 - Path b | Expectations → Side effects | 0.13 | <.001 | 0.133 | <.001 |
| Week 16 | 4 - Direct effect (c') | Trait anxiety → Side effects | 0.01 | 0.735 | 0.003 | .804 |
| Week 16 | 4 - Path b' | Expectations → Side effects | 0.13 | 0.001 | 0.131 | <.001 |

**Table S2**

Spearman rank correlations (r) between potential confounders and the primary study variables. Variables were considered classical confounders only if they showed a meaningful association with both the predictor (trait anxiety) and the outcome (experienced side effects). N = 104. DLQI = Dermatology Life Quality Index; PASI = Psoriasis Area and Severity Index; TEX = Treatment Expectation Questionnaire; GEEE = Generic Rating Scale for Treatment Experiences, Expectations, and Effects.

| **Variable** | **Variable** | **r** | **p** | **n** |
| --- | --- | --- | --- | --- |
| Age | Trait Anxiety | -0.10 | .292 | 104 |
| Age | Expectations (TEX, V1) | -0.21 | .033 | 104 |
| Age | Side Effects (GEEE, Week 4) | -0.32 | .001 | 104 |
| Age | Side Effects (GEEE, Week 16) | -0.13 | .193 | 103 |
| Sex (male = 1) | Trait Anxiety | -0.25 | .01 | 104 |
| Sex (male = 1) | Expectations (TEX, V1) | 0.07 | .473 | 104 |
| Sex (male = 1) | Side Effects (GEEE, Week 4) | -0.04 | .68 | 104 |
| Sex (male = 1) | Side Effects (GEEE, Week 16) | -0.01 | .942 | 103 |
| BMI | Trait Anxiety | 0.001 | .99 | 103 |
| BMI | Expectations (TEX, V1) | 0.17 | .083 | 103 |
| BMI | Side Effects (GEEE, Week 4) | 0.11 | .257 | 103 |
| BMI | Side Effects (GEEE, Week 16) | 0.1 | .341 | 102 |
| PASI | Trait Anxiety | -0.09 | .346 | 104 |
| PASI | Expectations (TEX, V1) | -0.10 | .292 | 104 |
| PASI | Side Effects (GEEE, Week 4) | -0.002 | .98 | 104 |
| PASI | Side Effects (GEEE, Week 16) | -0.33 | .001 | 103 |
| DLQI | Trait Anxiety | 0.39 | <.001 | 104 |
| DLQI | Expectations (TEX, V1) | 0.25 | .01 | 104 |
| DLQI | Side Effects (GEEE, Week 4) | 0.06 | .577 | 104 |
| DLQI | Side Effects (GEEE, Week 16) | 0.02 | .817 | 103 |

**Table S3**

Sensitivity analysis additionally controlling for age. Unstandardized regression coefficients (β) and p-values are shown for the four-step Baron and Kenny (1986) mediation model at Week 4 and Week 16, comparing the primary analysis (covariate: dose group only) with the age-adjusted analysis (covariates: dose group and age). Age was included as an additional covariate due to its moderate correlation with experienced side effects at Week 4 (r = −.32).

| **Timepoint** | **Step** | **Path** | **Main β (N=104)** | **Main p** | **Age-adjusted β** | **Age-adjusted p** |
| --- | --- | --- | --- | --- | --- | --- |
| Week 4 | 1 - Total effect (c) | Trait anxiety → Side effects | 0.04 | 0.007 | 0.038 | .014 |
| Week 4 | 2 - Path a | Trait anxiety → Expectations | 0.13 | <.001 | 0.125 | .001 |
| Week 4 | 3 - Path b | Expectations → Side effects | 0.30 | 0.008 | 0.126 | .002 |
| Week 4 | 4 - Direct effect (c') | Trait anxiety → Side effects | 0.03 | 0.098 | 0.025 | .11 |
| Week 4 | 4 - Path b' | Expectations → Side effects | 0.12 | 0.003 | 0.104 | .012 |
| Week 16 | 1 - Total effect (c) | Trait anxiety → Side effects | 0.02 | 0.116 | 0.021 | .154 |
| Week 16 | 2 - Path a | Trait anxiety → Expectations | 0.13 | <.001 | 0.125 | .001 |
| Week 16 | 3 - Path b | Expectations → Side effects | 0.13 | <.001 | 0.127 | .001 |
| Week 16 | 4 - Direct effect (c') | Trait anxiety → Side effects | 0.01 | 0.735 | 0.005 | .763 |
| Week 16 | 4 - Path b' | Expectations → Side effects | 0.13 | 0.001 | 0.123 | .002 |

**Table S4**

Supplementary mediation analyses using state anxiety (STADI-S) as the primary predictor in place of trait anxiety. State anxiety was assessed at Visit 1 (baseline) and Visit 2 (prior to first injection). The mediator (side effect expectations, TEX-Q, Visit 1) and outcomes (square root-transformed GEEE scores at Week 4 and Week 16) were identical to the primary analysis. Unstandardized regression coefficients (β), standard errors (SE), and p-values are reported for all four Baron and Kenny (1986) mediation steps. Dose group was included as a covariate throughout.

| **Timepoint** | **Predictor** | **Step** | **Path** | **β** | **SE** | **p** |
| --- | --- | --- | --- | --- | --- | --- |
| Week 4 | STADIS_A_v1 | 1 - Total effect (c) | STADIS_A_v1 → Side effects | 0.035 | 0.017 | .041 |
| Week 4 | STADIS_A_v1 | 2 - Path a | STADIS_A_v1 → Expectations | 0.182 | 0.037 | <.001 |
| Week 4 | STADIS_A_v1 | 3 - Path b | Expectations → Side effects | 0.147 | 0.039 | <.001 |
| Week 4 | STADIS_A_v1 | 4 - Direct effect (c') | STADIS_A_v1 → Side effects | 0.01 | 0.018 | .578 |
| Week 4 | STADIS_A_v1 | 4 - Path b' | Expectations → Side effects | 0.136 | 0.044 | .003 |
| Week 16 | STADIS_A_v1 | 1 - Total effect (c) | STADIS_A_v1 → Side effects | 0.023 | 0.015 | .139 |
| Week 16 | STADIS_A_v1 | 2 - Path a | STADIS_A_v1 → Expectations | 0.182 | 0.037 | <.001 |
| Week 16 | STADIS_A_v1 | 3 - Path b | Expectations → Side effects | 0.132 | 0.035 | <.001 |
| Week 16 | STADIS_A_v1 | 4 - Direct effect (c') | STADIS_A_v1 → Side effects | -0.001 | 0.016 | .933 |
| Week 16 | STADIS_A_v1 | 4 - Path b' | Expectations → Side effects | 0.134 | 0.04 | .001 |
| Week 4 | STADIS_A_v2 | 1 - Total effect (c) | STADIS_A_v2 → Side effects | 0.046 | 0.016 | .006 |
| Week 4 | STADIS_A_v2 | 2 - Path a | STADIS_A_v2 → Expectations | 0.148 | 0.038 | <.001 |
| Week 4 | STADIS_A_v2 | 3 - Path b | Expectations → Side effects | 0.147 | 0.039 | <.001 |
| Week 4 | STADIS_A_v2 | 4 - Direct effect (c') | STADIS_A_v2 → Side effects | 0.028 | 0.017 | .098 |
| Week 4 | STADIS_A_v2 | 4 - Path b' | Expectations → Side effects | 0.122 | 0.042 | .005 |
| Week 16 | STADIS_A_v2 | 1 - Total effect (c) | STADIS_A_v2 → Side effects | 0.011 | 0.015 | .467 |
| Week 16 | STADIS_A_v2 | 2 - Path a | STADIS_A_v2 → Expectations | 0.148 | 0.038 | <.001 |
| Week 16 | STADIS_A_v2 | 3 - Path b | Expectations → Side effects | 0.132 | 0.035 | <.001 |
| Week 16 | STADIS_A_v2 | 4 - Direct effect (c') | STADIS_A_v2 → Side effects | -0.01 | 0.015 | .516 |
| Week 16 | STADIS_A_v2 | 4 - Path b' | Expectations → Side effects | 0.142 | 0.038 | <.001 |

**Table S5**

Model assumption diagnostics for all regression models included in the primary mediation analyses. For each model, results of the Shapiro-Wilk test (W, p) for normality of residuals, the Breusch-Pagan test (BP, p) for homoscedasticity, and the maximum variance inflation factor (VIF) for multicollinearity are reported.

| **Timepoint** | **Step** | **Path** | **Shapiro-Wilk W** | **Shapiro-Wilk p** | **Breusch-Pagan BP** | **Breusch-Pagan p** | **Max VIF** |
| --- | --- | --- | --- | --- | --- | --- | --- |
| Week 4 | 1 - Total effect (c) | Trait anxiety → Side effects | 0.915 | <.001 | 2.552 | .279 | 1.019 |
| Week 4 | 2 - Path a | Trait anxiety → Expectations | 0.986 | .338 | 4.32 | .115 | 1.019 |
| Week 4 | 3 - Path b | Expectations → Side effects | 0.82 | <.001 | 0.006 | .997 | 1.001 |
| Week 4 | 4 - Direct effect (c'b') | Anxiety + Expectations → Side effects | 0.954 | .001 | 6.225 | .101 | 1.149 |
| Week 16 | 1 - Total effect (c) | Trait anxiety → Side effects | 0.813 | <.001 | 11.893 | .003 | 1.013 |
| Week 16 | 2 - Path a | Trait anxiety → Expectations | 0.986 | .338 | 4.32 | .115 | 1.019 |
| Week 16 | 3 - Path b | Expectations → Side effects | 0.92 | <.001 | 20.054 | <.001 | 1.002 |
| Week 16 | 4 - Direct effect (c'b') | Anxiety + Expectations → Side effects | 0.921 | <.001 | 24.031 | <.001 | 1.165 |
